# Implementation of prognostic machine learning algorithms in paediatric chronic respiratory conditions: a scoping review

**DOI:** 10.1136/bmjresp-2021-001165

**Published:** 2022-03-16

**Authors:** Nicole Filipow, Eleanor Main, Neil J Sebire, John Booth, Andrew M Taylor, Gwyneth Davies, Sanja Stanojevic

**Affiliations:** 1 UCL Great Ormond Street Institute of Child Health, University College London, London, UK; 2 Population, Policy and Practice Research and Teaching Department, UCL Great Ormond Street Institute of Child Health, University College London, London, UK; 3 GOSH NIHR BRC, Great Ormond Street Hospital for Children, London, UK; 4 Institute of Cardiovascular Science, University College London, London, UK; 5 Community Health and Epidemiology, Dalhousie University, Halifax, Nova Scotia, Canada

**Keywords:** bronchiectasis, cystic fibrosis, paediatric asthma, paediatric lung disaese

## Abstract

Machine learning (ML) holds great potential for predicting clinical outcomes in heterogeneous chronic respiratory diseases (CRD) affecting children, where timely individualised treatments offer opportunities for health optimisation. This paper identifies rate-limiting steps in ML prediction model development that impair clinical translation and discusses regulatory, clinical and ethical considerations for ML implementation. A scoping review of ML prediction models in paediatric CRDs was undertaken using the PRISMA extension scoping review guidelines. From 1209 results, 25 articles published between 2013 and 2021 were evaluated for features of a good clinical prediction model using the Transparent Reporting of a multivariable prediction model for Individual Prognosis Or Diagnosis (TRIPOD) guidelines.

Most of the studies were in asthma (80%), with few in cystic fibrosis (12%), bronchiolitis (4%) and childhood wheeze (4%). There were inconsistencies in model reporting and studies were limited by a lack of validation, and absence of equations or code for replication. Clinician involvement during ML model development is essential and diversity, equity and inclusion should be assessed at each step of the ML pipeline to ensure algorithms do not promote or amplify health disparities among marginalised groups. As ML prediction studies become more frequent, it is important that models are rigorously developed using published guidelines and take account of regulatory frameworks which depend on model complexity, patient safety, accountability and liability.

## Introduction

The rapidly expanding field of machine learning (ML) has created widespread promise in healthcare for the diagnosis, prognosis and management of disease to ultimately enrich personalised medicine. ML is a broad field that uses statistics and algorithms to acquire knowledge from existing data, with the aim of predicting a future outcome for a set of similar circumstances, and the opportunity for an ongoing process of updating and fine tuning when new data are available. A rapid expansion in the application of ML in medicine has been fuelled by vast amounts of data captured through clinical records, imaging, diagnostic investigations, patient registries and more recently electronic health records (EHRs) and wearable devices. As automated data capture becomes more widespread in routine care, so too does the potential for ML models to diagnose disease or predict disease trajectories.

### Machine learning

**Figure 1:**
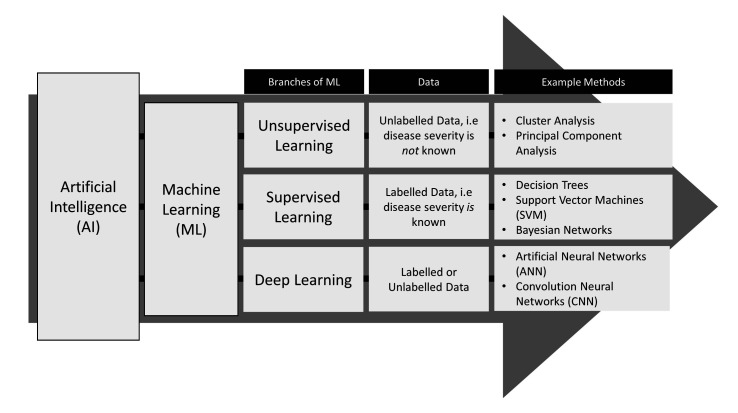
Branches of machine learning.

A branch of artificial intelligence, ML uses algorithms to identify patterns in often large and complex datasets that traditional statistical methods can have difficulty uncovering.^1^ Broadly, ML is separated into supervised, unsupervised or deep learning; each is employed depending on the objective of the analysis and the information presented in the data (figure 1).^2^ Supervised methods form prediction models based on data with labelled outcomes, for example, when the disease severity of patients is known. Unsupervised methods are used to identify the shared characteristics between similar groups of data where the outcomes are not labelled or defined, for example, to identify clinically meaningful subgroups of disease when the relative disease severities of patients are unknown. Deep learning may be supervised or unsupervised and uses artificial neural networks (ANNs) to learn from data. ANNs are complex models that use many interconnected layers of processing units, termed neurons, which extract levels of information from raw data to generate a set of rules for predictions.^3^

### Predicting clinical outcomes in paediatric chronic respiratory diseases

ML predictive algorithms are particularly attractive within the field of chronic respiratory diseases (CRD), which present with heterogeneous clinical outcomes from diagnosis across the life course. In CRDs that affect children such as asthma, cystic fibrosis (CF), primary ciliary dyskinesia (PCD), bronchopulmonary dysplasia (BPD) and children’s interstitial lung disease (chILD), the prediction of clinical outcomes is especially important, where timely individualised treatment regimes offer opportunities for health maintenance before symptoms of the disease become severe and irreversible.^4^ CRDs in children often involve longitudinal follow-up over multiple years with complex outcomes from clinical encounters which may be captured repeatedly through patient registries, cohort studies, or EHRs. These large datasets have driven the development of ML algorithms to predict likelihood of unfavourable clinical outcomes common in paediatrics such as respiratory exacerbation, hospitalisation, or accelerated lung function decline, with the aim of supporting early treatment decisions to prevent severe outcomes such as lung transplant or death.^5 6^ The adoption of ML predictive models in clinical care is rare however, which is discouraging given the increase of ML publications in respiratory medicine in the last decade.^7^ A series of recent reviews in other disease areas has highlighted inaccuracies and failures in reporting standards of prognostic models generally as the major constraint to clinical translation.^8–10^

### Objectives

While opportunities exist for ML prediction models to impact clinical care, challenges to implementation remain a barrier to clinical use. To explore the gap between model development and clinical application specific to CRDs affecting children, we carried out a scoping review of the available literature to evaluate the reporting of ML prediction models and identify the rate-limiting steps in model development that impair clinical translation. We further discuss regulatory, clinical and ethical considerations for implementation and the future opportunities for EHRs to influence ML prediction models in clinical care.

## Methods

### Overview

We carried out a scoping review using the Preferred Reporting Items for Systematic reviews and Meta-Analyses extension for scoping reviews guidelines^11^ to identify prognostic ML algorithms in CRDs that affect children, including but not limited to CF, bronchiectasis, asthma, PCD, BPD and chILD. The purpose of the review was not to provide a summary of models in specific diseases, but rather to investigate the rate limiting steps to clinical implementation of ML predictive models generally across paediatric CRDs, which have in common similar predictors and outcomes.

To identify barriers to clinical implementation within model development, relevant ML models were evaluated with reference to the key recommendations for model reporting specific for respiratory, sleep and critical care studies, summarised below.^12^ These metrics were summarised from the published guidelines for the Transparent Reporting of a multivariable prediction model for Individual Prognosis Or Diagnosis (TRIPOD).^13 14^

### Search criteria

A search for published articles was performed in the Medline database using a previously curated list of phrases to identify prediction studies,^15^ and included the updated phrase suggestions.^16^ To filter for studies that used ML, the following MeSH terms and keywords were included: (Unsupervised Machine Learning/ or unsupervised learning.mp.) or (machine learning.mp. or Machine Learning/) or (artificial intelligence.mp. or Artificial Intelligence/) or (Supervised Machine Learning/ or supervised learning.mp.) or (deep learning.mp. or Deep Learning/) or (Neural Networks, Computer/ or neural network*.mp.) or ((cluster analysis or clustering).mp. or Cluster Analysis/) or ((support vector machine or SVM).mp. or Support Vector Machine/) or random forest*.mp. or (decision tree*.mp. or Decision Trees/) or Bayesian.mp.

Respiratory MeSH terms and keywords included (cystic fibrosis.mp. or Cystic Fibrosis/) or (Asthma/ or asthma.mp.) or (Bronchiectasis/ or bronciectasis.mp.) or (Bronchopulmonary Dysplasia.mp. or Bronchopulmonary Dysplasia/) or (primary ciliary dyskinesia.mp. or Ciliary Motility Disorders/) or (interstitial lung disease.mp. or Lung Diseases, Interstitial/) or (chronic respiratory disease or chronic respiratory illness or chronic respiratory condition).mp.

Paediatric studies were identified from the patient ages in the study data rather than included as a search term to not exclude articles that did not specifically mention paediatrics. The search was limited to publications in the past decade (2011–15 October 2021), since it was anticipated that most ML prediction models would have been recently published given the rise in ML studies in respiratory medicine in the past decade.^7^ Furthermore, EHR systems were not implemented widely into healthcare systems prior to 2010.^17^ Any subsequent related studies of relevant articles were searched for to ensure all aspects of model development and validation were captured.

Articles were excluded from review based on the following criteria: (1) not a primary journal article, (2) irrelevant (ie, in vitro studies, pharmacokinetic models, ML model not developed, not a CRD), (3) diagnostic or disease differentiation models, (4) descriptive models, (5) not predictive of clinical outcomes (ie, predictive of cost of care), (6) did not use primarily paediatric data, (7) did not report the age of study participants. The initial search results were filtered through a title search, and potential articles were further screened through a review of abstracts and full text.

### Evaluating ML prediction studies

Using hallmarks of ML prediction studies identified in Leisman *et al*,^12^ models were evaluated for their reporting of metrics that infer features of a good clinical prediction model: generalisability, biasedness, interpretability, replicability and clinical performance^13 18–20^ (table 1).

**Table 1:** Key reporting elements evaluated in this scoping review to infer features of a good clinical prediction model

Features	Definition	Key reporting elements*
Generalisability	How well the model works in populations external to the study population, and as such can be used to infer performance in a clinical setting	Data source Participants Validation (internal/external)
Biasedness	Occurs when certain elements are more heavily weighted than others, or with inconsistency or subjectivity in defining the outcome.	Missing Data Outcomes
Interpretability	How well the model is understood by clinicians	Predictors
Replicability	The ability to replicate the model in the same or independent population	Model specification Model structure
Performance	Whether the model provides benefit to patients	Prospective study Randomised controlled trial

*Transparent Reporting of a multivariable prediction model for Individual Prognosis Or Diagnosis guidelines summarised in Leisman *et al.*^12^

Generalisability was assessed through investigation of study characteristics including the location, data source, number of centres, dates of investigation, patient characteristics, as well as evidence of internal and/or external validations. To investigate potential sources of bias, the handling of missing data, sample size and the definition of the outcome was noted. Interpretability was inferred through methods of predictor selection, numbers of predictors, as well as any methods to meaningfully describe the resulting model. Replicability was inferred if the model structure/specificity was provided through equations or shared code.

Traditional measures of model performance involve a range of metrics that assess how well the model classifies data compared with the labelled classifications during internal and external validations, such as area under the receiver operator curve, specificity/sensitivity, accuracy, or precision–recall curves.^12^ However, high performance by these measures does not inevitably represent clinical efficacy or patient benefit.^18^ As such, this review focused on whether a prognostic study in a clinical setting or a randomised controlled trial (RCT) has been carried out to evaluate clinical performance of the ML model.

## Results

### Study selection

**Figure 2:**
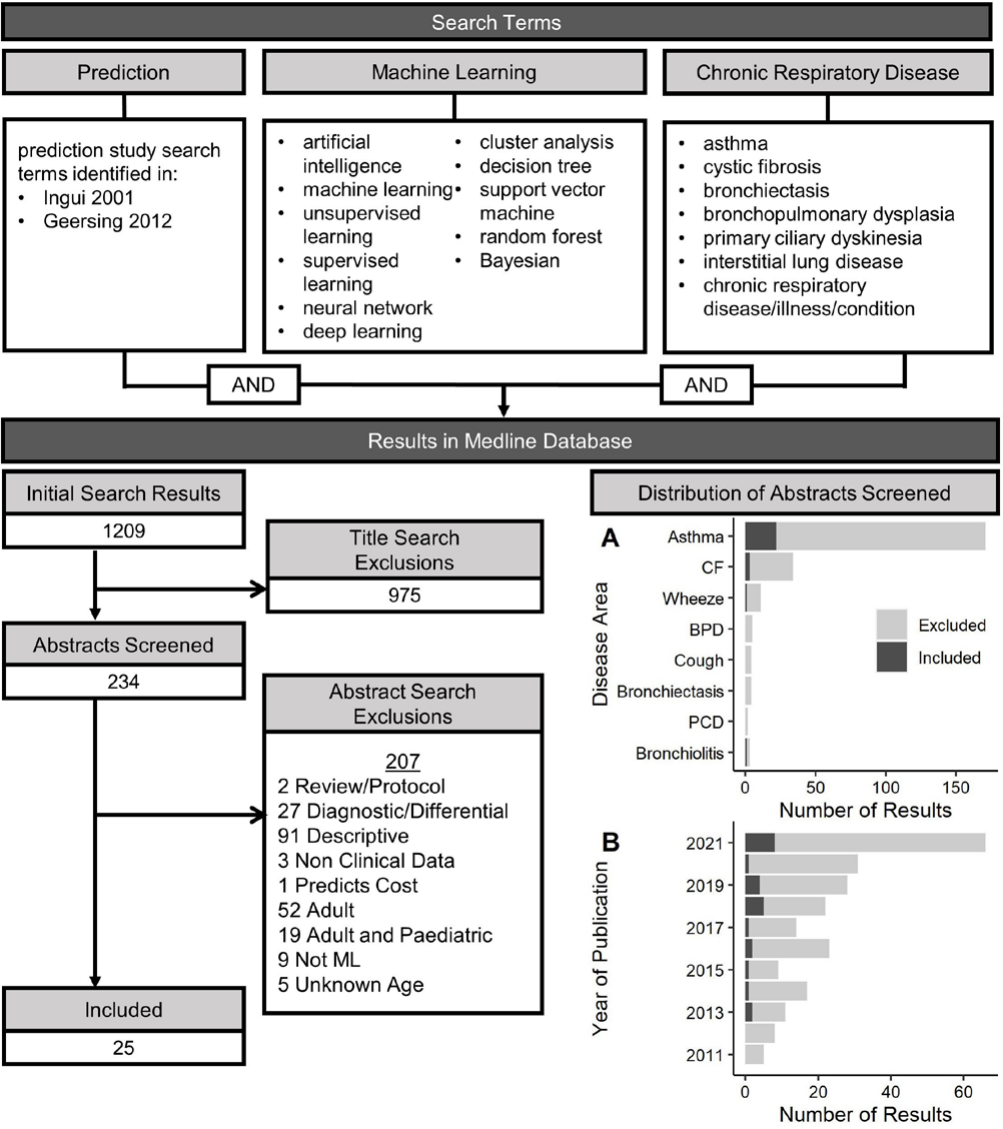
Relevant search terms and results for a literature review identifying studies that used machine learning (ML) methods to develop prediction models of clinical outcomes in chronic respiratory diseases affecting children. Results were filtered through a title review, and abstracts were further screened with the exclusion criteria to identify relevant ML models in paediatrics. (A) Distribution of diseases and (B) years of publications identified in the search process; light grey=articles flagged as relevant through a title search, dark grey=articles selected for review based on the exclusion criteria. BPD, bronchopulmonary dysplasia; CF, cystic fibrosis; PCD, primary ciliary dyskinesia.

A flow chart of the scoping review process is displayed in figure 2. There were 1209 results, 243 abstracts were screened and 25 articles were included in the review, which are summarised in table 2.

**Table 2:** Summary of ML prediction studies reviewed

Author	Disease	Data source	Centres	Study dates	Age range	Primary outcome(s)	Missing data	ML method (best model)	Validation		Prospective study/RCT
									Internal	External	
Hogan *et al* 2021^21^	Asthma	Registry (National Database)	NR	2013	5–18	Hospitalisation	Complete case analysis	ANN	Yes	No	No
Filipow *et al* 2021^22^	CF	Registry (Regional Database)	1	2000–2018	2–18	Hospitalisation, exacerbation	Complete case analysis	Cluster Analysis	Yes	Yes	No
Bose *et al* 2021^30^	Asthma	Registry (EHR)	30+	2005–2016	2–5	Asthma persistence	Mixed	XGBoost	Yes	No	No
van Horck *et al* 2021^38^	CF	Study data	3	NR	5–18	Exacerbation	NR	Random Forest	No	No	No
Raita *et al* 2021^25^	Bronchi-olitis	Study data	17	2011–2014	<1	Wheeze, asthma	Imputed	Cluster Analysis	No	No	No
Sills *et al* 2021^26^	Asthma	Registry (EHR)	5	2009–2013	2–21	Hospitalisation	Complete case analysis	AutoML	Yes	No	No
Seol *et al* 2021^29^	Asthma	NR	1	NR	<18	Exacerbation	NR	Naïve Bayes	Yes	No	Yes
Lovrić *et al* 2021^42^	Asthma	Study data	1	NR	2–22	Response to treatment	Imputed	AdaBoost	Yes	No	No
Caparrós-Martín *et al* 2020^44^	CF	Study data	NR	NR	2.5–4.4	Lung damage (quantified CT)	NR	Cluster Analysis	No	No	No
Wang *et al* 2019^31^	Asthma	Study data	8	1993–1995	5–12	Asthma remission	Complete case analysis	Decision Tree	No	No	No
Messinger *et al* 2019^32^	Asthma	Registry (EHR)	1	2016–2017	2–18	Paediatric Asthma Score	Complete case analysis	ANN	Yes	No	No
Khasha *et al* 2019^45^	Asthma	Study data	1	NR	>5	Asthma control	Imputed	Ensemble Learning	No	No	No
Goto *et al* 2019^33^	Asthma	Registry (National Database)	NR	2007–2015	2–14 (IQR)	Critical care (admission to intensive care unit or death), hospitalisation	Imputed	Decision Tree	Yes	No	No
Patel *et al* 2018^34^	Asthma	Registry (EHR)	2	2012–2015	2–18	Hospitalisation	Complete case analysis	Gradient Boosting	Yes	No	No
Ross *et al* 2018^35^	Asthma	Study data	8	1993–1995	5–12	Asthma control	Imputed	Predictor Pursuit	Yes	No	No
Spyroglou *et al* 2018^40^	Asthma	Study data	1	2008–2016	1–14.5	Exacerbation	NR	Bayesian	Yes	No	No
Huffaker *et al* 2018^27^	Asthma	Study data	1	2016–2017	5–17	Exacerbation	Complete case analysis	Random Forest	Yes	No	No
Shin *et al* 2018^28^	Asthma	Registry (EHR)	1	2016	6–18	Hospitalisation	NR	Random Forest	Yes	No	No
Das *et al* 2017^36^	Asthma	Registry (EHR)	1	2013–2014	≤18	Frequent emergency department visits	NR	Logistic Regression	Yes	No	No
Pite *et al* 2016^39^	Wheeze	Study data	1	1993–2006	≤7	Asthma development in adolescence	Complete case analysis	Cluster Analysis	No	No	No
Van Vliet *et al* 2016^43^	Asthma	Study data	1	NR	6–18	Asthma control	NR	Random Forest	Yes	No	No
Luo *et al* 2015^37^	Asthma	Study data	4	2011–2012	2–18	Asthma control 1 week prior	NR	Multiboost with Decision Stumps	Yes	No	No
Howrylak *et al* 2014^23^	Asthma	Study data	8	1993–1995	5–12	Exacerbation	Imputed	Cluster Analysis	No	No	No
Farion *et al* 2013^24^	Asthma	Registry (clinical records)	1	2000–2004	1–17	Exacerbation severity	Imputed	Naïve Bayes	Yes	No	Yes
Robroeks *et al* 2013^41^	Asthma	Study data	1	NR	6–16	Exacerbation	NR	SVM	Yes	No	No

ANN, artificial neural network; EHR, electronic health record; NR, not reported; RCT, randomised controlled trial; SVM, support vector machine.

The studies selected for review were published between 2013 and 2021; 72% were published since 2018 (figure 2). Most of the studies were related to asthma (80%), with few in CF (12%), bronchiolitis (4%) and childhood wheeze (4%). While a small number of studies using ML were identified in BPD, PCD, chronic cough and bronchiectasis, they were either diagnostic or disease differentiation ML models, or were carried out in adults and were excluded. The majority of studies used disease exacerbation or hospitalisation as an outcome, with other predictions including risk of lung damage (through quantification of imaging with CT), development of comorbidities (eg, developing asthma in childhood wheeze), disease-specific measures such as asthma control, or positive clinical outcomes such as asthma remission or response to treatment. One study examined risk of critical care, which was defined as admission to ICU or death; otherwise, risk of death or lung transplant was not assessed as a primary outcome which was expected, given their rare occurrence in paediatrics.

A range of ML algorithms were used, with more studies using supervised (72%) over unsupervised (20%) and deep learning (8%) methods. Some studies employed multiple ML methods to identify the optimal model while others focused on the development of a single model. Random forest was the most widely used supervised method (n=10), followed by decision trees (n=5), Bayesian models (n=4), support vector machines (n=4), Lasso (n=3), various boosting methods (n=3), and combined models (ie, autoML, ensemble learning, predictor pursuit), where predictions are made from multiple sequential methods (n=3). ANNs were the only deep learning methods used and cluster analysis was the only unsupervised method. Many of the descriptive studies excluded from review used cluster analysis to define the characteristics of subgroups of disease, without predicting future outcomes.

### Generalisability

**Figure 3:**
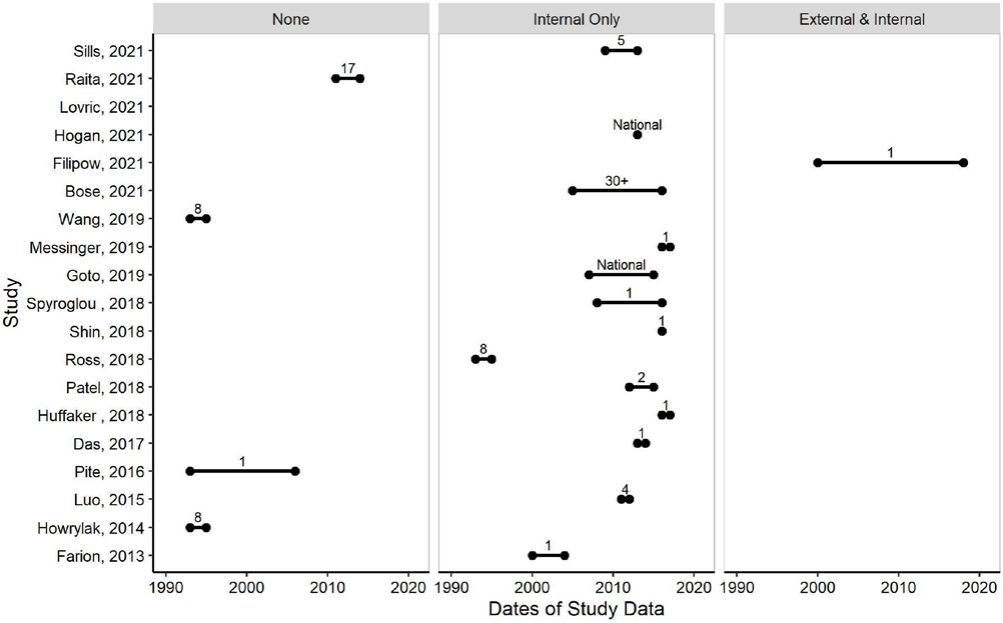
Evaluating the generalisability of machine learning (ML) models through investigating dates of study data (x-axis) for each study (y-axis), evidence of validations (left=no validations, middle=internal validation only, right=external and internal validation), and number of centres (labelled on figure; ‘National’=If a national registry/database was used and the number of centres within were not reported). Only studies that reported each of the data and patient metrics are displayed (n=19/25).

There were 19 studies (76%) that reported each of the data and patient metrics used to infer generalisability (figure 3). Often the data were described from previous studies but it was not always clear if the original data exclusions also applied to the present study. Clarity on these details should be included in the main text. Most studies originated from a single centre (52%) rather than multicentre or a national database (44%). There were more studies with data from North America (68%)^21–37^ than Europe (24%),^38–43^ Australia (4%),^44^ or the Middle East (4%).^45^ Data collected during studies (ie, cross-sectional, longitudinal cohort) were the most common sources of data (56%),^23 25 27 31 35 37–45^ followed by registry data from either routine EHR (24%),^26 28 30 32 34 36^ regional or national databases (12%),^21 22 33^ or clinical records (4%).^24^ One study did not report the source of data for model development (4%).^29^

Participant ages within a study ranged from infants less than a year old, to 2–22 years. Dates of study data ranged from 1993 to 2019, 6 studies (24%)^29 38 41 43–45^ did not report any study dates. There was no evidence of any model being updated over time. Year of publication did not always correlate with years of study data.

There were 18 studies (72%)^21 22 24 26–30 32–37 40–43^ that carried out an internal validation. This was most often accomplished by splitting the dataset into a test and training set. Only a single study (4%)^22^ carried out an external validation in geographically different data (figure 3).

### Bias

Sample sizes ranged considerably across studies, from small scale studies (n=49 people) to larger analyses (n=52 037 people). It was often not clear whether large-scale studies included data of repeated measures, or if they were independent records. In handling missing values, 32% of studies used complete case analysis,^21 22 26 27 31 32 34 39^ 28% imputed missing values,^23–25 33 35 42 45^ and one used a combination of both^30^; however, 36% of studies did not define any explicit methods.^28 29 36–38 40 41 43 44^ In defining outcomes, proxy measures were often used, for example exacerbation was often recorded as requirement of a medication, which can be biased towards clinician or centre treatment preferences.

### Interpretability

The number of predictors ranged from 9 to 648. Studies using large numbers of predictors (n>50) did not typically rely on any variable reduction techniques or they did not describe if or which variables were included in the final prediction model if variable reduction was considered.^30 35 43^ These models are uninterpretable as it is unknown which of the hundreds of variables influenced clinically relevant poor outcomes for a particular person. Studies using smaller numbers often used clinical knowledge to select variables,^22 23 25 29 31–33 36^ excluded those with high missingness,^24 35 42^ or used various statistical techniques to ensure included variables were clinically relevant,^38 39 41 45^ which may allow for more interpretability.

### Repeatability

None of the studies shared any code or equations for their predictive models.

### Clinical performance

Two studies carried out prospective studies to assess the performance of a ML model in a clinical setting. One study assessed the accuracy of a naïve Bayes model compared with both a standard score and physician decisions in a prospective study to predict severity of asthma exacerbation in the emergency department. The naïve Bayes performed with less accuracy than both.^24^

In an RCT, the second study provided an asthma exacerbation prediction model to physicians in the intervention group, while standard care was maintained in the control group. There was no difference in prevalence of exacerbation within 1 year for patients in either group, although the physicians in the intervention group had a reduced time in reviewing EHRs for asthma management.^29^

## Discussion

The 25 prognostic ML studies assessed in this scoping review were overwhelmingly focused on asthma and the majority were supervised models. The studies were mainly limited by a lack of validation or prospective study, and the absence of equations or code for replication, which are major steps required for clinical implementation. Some recent studies used data from 1 to 2 decades ago, which may have limited relevance to current populations for which treatments and care have changed. Some of the models were opaque, uninterpretable models that used high numbers of predictors and did not explain the resulting predictions. This is especially important in healthcare since a clinician needs to know not only who is at risk, but also what they can do to change the outcome.

A large proportion of studies did not report on the handling of missing data, which does not provide transparency to evaluate whether sample populations are under-represented, for example, towards those who are sicker and have more data. Smaller datasets were typically derived from research studies, where there is greater control over the variables collected or the inclusion criteria for the study. However, ML methods were typically developed for large datasets, and studies using national/regional databases, EHRs, or data from daily home monitoring benefit from large samples likely more representative of wider populations.

External validations are necessary to understand the generalisability of the predictions; however, only one was conducted. In the study, similar clusters of children with CF developed from data in Canada were identified in data from the UK, providing evidence for the generalisability of the model.^22^ Internal validations were frequent, but their performance relies heavily on the definition of the outcome. If the outcome is somewhat subjectively captured, for example, prescription of medication, the resulting predictions are biased towards the subjective. This is highlighted in the two prospective studies that identified no patient benefit despite good model performance during development.^24 29^ If the models are trained on data where the outcome is influenced by clinician decision, it is unsurprising that the models would not outperform a clinician. While these models may benefit areas of healthcare such as easing/increasing clinician workflow, objectively captured outcomes such as chest imaging, lung function, or physiological data may result in models with greater patient benefit.

This scoping review was limited in that the studies were not assessed with the full TRIPOD guidelines, and bias and clinical applicability were not assessed with the full Prediction model Risk Of Bias ASsessment Tool^46^ guidelines. A summarised reporting checklist was instead used, which investigated the articles at an overarching level rather than a granular level to identify key themes. Even without detailed assessment using the full reporting checklists, the summarised checklist revealed that studies still largely failed to report on or carry out key metrics, and thus more granular investigation at this point was not required to identify shortcomings in model reporting. Development of ML prediction models is still an unexplored area of research in paediatric CRDs other than asthma, highlighted here by a lack of studies identified in other respiratory conditions. As research into these areas continues, and as ML prediction studies in paediatric CRDs are becoming more frequent (72% published since 2018), it is important that the models are rigorously developed. A quality assessment tool for artificial intelligence-centered diagnostic studies is currently being developed, and combined with the TRIPOD guidelines for prediction studies will be useful for designing future ML prediction models with clinical implications.^47^

### Further considerations

**Figure 4:**
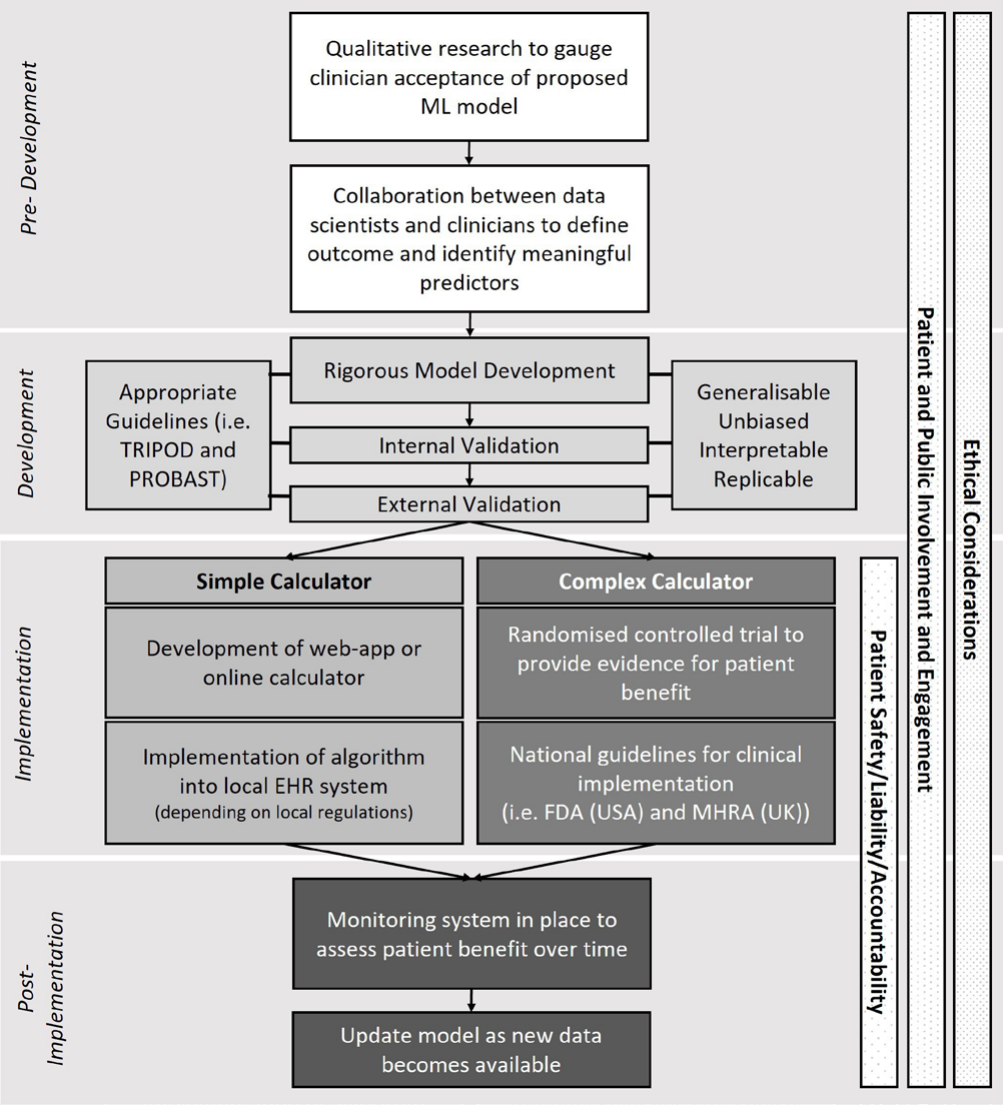
Hypothetical framework for developing machine learning (ML) prediction models in healthcare. EHR, electronic health record; FDA, Food & Drugs Administration; MHRA, Medicines and Healthcare products Regulatory Agency; PROBAST, Prediction model Risk Of Bias ASsessment Tool; TRIPOD, Transparent Reporting of a multivariable prediction model for Individual Prognosis Or Diagnosis.

The lack of model implementation is a point of discussion in healthcare generally, and in addition to model development and reporting require regulatory, clinical and ethical frameworks.^18 48–51^ A hypothetical pathway for ML model development using these frameworks is summarised in figure 4.

#### Regulatory

The level of regulation and approval required for a prediction model can depend on its complexity, where more complicated, uninterpretable models are classed as a medical device and must be approved by relevant governing bodies, such as the Food & Drugs Administration (FDA) in the USA, or the Medicines and Healthcare products Regulatory Agency in the UK. A recent online database suggests that 64 AI/ML-based models have been FDA approved since the first one in 2016, which are predominantly within the fields of radiology and cardiology.^52^ Alternatively, simple models that prevent overfitting while improving interpretability may require less regulation if classed as a simple calculator. Depending on local regulations, these simple models may be employed using an app, online calculator, or hosted on platforms such as Programmable Interface for Statistical & Simulation Models (https://resp.core.ubc.ca/research/Specific_Projects/PRISM).

Regulatory pathways for AI-based and ML-based medical devices in the USA and Europe are outlined in Muehlematter *et al*.^53^ Generally, prospective studies and RCTs are the standards used by regulators to provide evidence for clinical decision-making.^19^ However, RCTs are time consuming and costly, which may explain why few were identified in this review. It has been suggested that observational real-world data from EHRs may be adequate to evaluate the performance of a ML model, and that while useful, prognostic studies and RCTs should not be solely relied on to bring ML to clinical care.^54 55^ Conversely, there is discussion that observational studies are less rigorous and have discrepant results to RCTs and should never be used to infer patient benefit.^56^ However, as EHRs and big data in healthcare accumulate and become increasingly representative of wider populations, it seems appropriate that methods to evaluate clinical effectiveness from observational data are given due consideration and acknowledged as a valuable resource complementary to RCTs. Appropriate design and methodology relating to evaluation of ML models in any RCT to evaluate their clinical utility will be an important discussion moving forward, and mutually agreed on guidelines by regulators and clinicians for model evaluation in EHR studies is necessary.

Patient safety, accountability and liability are further major considerations for implementation. A recent review suggested that the allocation of responsibility in ML models is not clear, and stronger guidelines are necessary to understand which stakeholders are responsible should a ML model contribute to patient harm.^57^ Decision support tools, which aid clinicians in their assessment of disease severity through associated risks, may require less accountability than decision making tools, where the model becomes automated and suggests or delivers treatments depending on thresholds of biomarkers or symptoms.^48^ Decision support tools are more likely to be fully realised in the short term over decision-making tools, since a clinician still acts as the final decision maker and is ultimately responsible. Without clearer regulations surrounding accountability and liability, and clearer frameworks for determining patient safety and benefit of ML models, the potential for implementation of decision-making tools is yet to be fully realised given the high risk of an erroneous prediction.^58^

#### Clinical

Implementation also requires the confidence of clinicians, and clinician involvement during model development is essential. Especially in respiratory disease, prior research has generated ample knowledge on contributors of poor outcomes, which should not be ignored in model development or assessment. Combining clinical knowledge with ML may improve both performance and clinical trust in models, better facilitating their adoption in clinical care.

There is currently a lack of knowledge translation and implementation science between data scientists and clinicians, which are needed to be integrated into model development. Qualitative research may be necessary to gauge acceptance and potential utility of predictive models before they are developed.

#### Ethical

ML algorithms have been known to amplify or create health disparities among marginalised groups. Ethical concerns can arise at every step of ML model development, including the selection/funding of the problem, collection of data, definition of the outcome, algorithm development and algorithm monitoring post deployment.^59^ These issues can arise from inconsistencies in access to healthcare or under representation of certain groups in particular centres, which is reflected in the data used to train models. Including variables directly in the model to account for marginalised groups, such as gender or ethnicity, is not always the best practice and may perpetuate the biases. A review detailing a roadmap for responsible and ethical ML in healthcare is useful for addressing some of these concerns.^60^ Diversity, equity and inclusion should be considered at every step of ML model development.

### Opportunities with EHRs

The opportunity for ML to support clinical decisions has been pronounced through the adoption of EHR in healthcare systems.^61^ EHRs are often unstructured and inconsistently captured; however, they are a rich, real-world source of vast amounts of clinical data useful for uncovering meaningful patterns. Data infrastructure plays a key role in harnessing EHRs to enable the extraction, processing and analysis of large volumes of data. Feasibility and interoperability between data systems are important for this process, and standards such as fast healthcare interoperability resources (FHIR) should be considered (https://www.hl7.org/fhir/).

**Figure 5:**
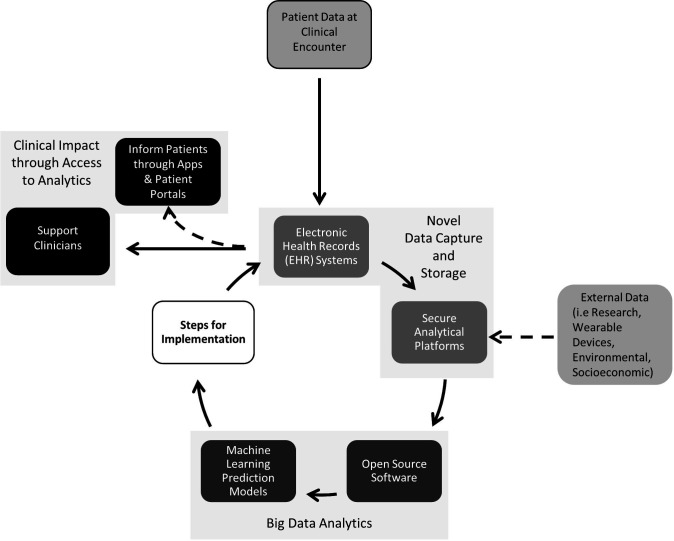
Opportunity for machine learning (ML) in clinical care with the availability of electronic health records (EHR) systems. Patient data at each clinical encounter is stored in secured databases or analytical platforms, which have the capacity or potential to be accessed by researchers. Open-source software in healthcare means collaboration is more feasible in developing ML models. Following the appropriate steps for regulation and implementation, the resulting algorithms can be fed back into EHR systems to calculate the risk of poor outcomes for incoming data from a new clinical encounter. This can be accessed by clinicians to support or inform clinical care and decision-making. The analytical approach also has the potential to merge data from external sources, such as research or wearable devices to improve model performance. Furthermore, patients can often now access their own EHR data through apps and online patient portals. This has the potential to display the results of individual calculated risks should it be considered appropriate and the appropriate regulatory or governance processes applied.

With appropriate infrastructure, a streamlined process between data capture, analytics and implementation can exist to predict outcomes for patient data at a new clinical encounter or visualise patient trajectories over time to support or inform clinical practice (figure 5). As EHR data grow large over time, the algorithms can and should be updated to reflect newer cohorts or include new information. The process is easily severed if steps for implementation are not considered or followed through, which risks an abundance of models that fail to be implemented into clinical practice. It is therefore necessary that models are developed to be generalisable, unbiased and interpretable with good clinical performance, and consider regulatory, clinical and ethical frameworks for implementation.

## Conclusions

The 25 prognostic ML algorithms in CRDs affecting children assessed in this scoping review were most notably limited by a lack of validations and replicability. For ML to enhance personalised medicine and influence clinical care, it is important that the models are rigorously developed and that the regulatory, clinical and ethical frameworks for implementation are considered at every step of the ML pipeline—from predevelopment to post implementation. This is especially important as EHRs become more widespread and facilitate the integration of ML algorithms directly into clinical care.

## References

[1] Bzdok D, Altman N, Krzywinski M. Points of significance: statistics versus machine learning. Nat Methods 2018;15:233–4. 30100822 10.1038/nmeth.4642 PMC6082636

[2] Erickson BJ, Korfiatis P, Akkus Z, et al. Machine learning for medical imaging. Radiographics 2017;37:505–15. 10.1148/rg.2017160130 28212054 PMC5375621

[3] Shahid N, Rappon T, Berta W. Applications of artificial neural networks in health care organizational decision-making: a scoping review. PLoS One 2019;14:e0212356. 10.1371/journal.pone.0212356 30779785 PMC6380578

[4] Hedlin G, Eber E, Aurora P, et al. Paediatric respiratory disease: past, present and future. Eur Respir J 2010;36:225–8. 10.1183/09031936.00085510 20675775

[5] Mlodzinski E, Stone DJ, Celi LA. Machine learning for pulmonary and critical care medicine: a narrative review. Pulm Ther 2020;6:67–77. 10.1007/s41030-020-00110-z 32048244 PMC7229087

[6] Khemasuwan D, Sorensen JS, Colt HG. Artificial intelligence in pulmonary medicine: computer vision, predictive model and COVID-19. Eur Respir Rev 2020;29:200181. 10.1183/16000617.0181-2020 33004526 PMC7537944

[7] Gonem S, Janssens W, Das N, et al. Applications of artificial intelligence and machine learning in respiratory medicine. Thorax 2020;75:695–701. 10.1136/thoraxjnl-2020-214556 32409611

[8] Wynants L, Van Calster B, Collins GS, et al. Prediction models for diagnosis and prognosis of covid-19: systematic review and critical appraisal. BMJ 2020;369:m1328. 10.1136/bmj.m1328 32265220 PMC7222643

[9] Simon-Pimmel J, Foucher Y, Léger M, et al. Methodological quality of multivariate prognostic models for intracranial haemorrhages in intensive care units: a systematic review. BMJ Open 2021;11:e047279. 10.1136/bmjopen-2020-047279 PMC8458313 34548347

[10] Wang W, Kiik M, Peek N, et al. A systematic review of machine learning models for predicting outcomes of stroke with structured data. PLoS One 2020;15:e0234722. 10.1371/journal.pone.0234722 32530947 PMC7292406

[11] Tricco AC, Lillie E, Zarin W, et al. PRISMA extension for scoping reviews (PRISMA-ScR): checklist and explanation. Ann Intern Med 2018;169:467–73. 10.7326/M18-0850 30178033

[12] Leisman DE, Harhay MO, Lederer DJ, et al. Development and reporting of prediction models: guidance for authors from editors of respiratory, sleep, and critical care journals. Crit Care Med 2020;48:623–33. 10.1097/CCM.0000000000004246 32141923 PMC7161722

[13] Moons KGM, Altman DG, Reitsma JB, et al. Transparent reporting of a multivariable prediction model for individual prognosis or diagnosis (TRIPOD): explanation and elaboration. Ann Intern Med 2015;162:W1–73. 10.7326/M14-0698 25560730

[14] Collins GS, Reitsma JB, Altman DG, et al. Transparent reporting of a multivariable prediction model for individual prognosis or diagnosis (TRIPOD): the TRIPOD statement. BMC Med 2015;13:1–10. 10.1186/s12916-014-0241-z 25563062 PMC4284921

[15] Ingui BJ, Rogers MA. Searching for clinical prediction rules in MEDLINE. J Am Med Inform Assoc 2001;8:391–7. 10.1136/jamia.2001.0080391 11418546 PMC130084

[16] Geersing G-J, Bouwmeester W, Zuithoff P, et al. Search filters for finding prognostic and diagnostic prediction studies in MEDLINE to enhance systematic reviews. PLoS One 2012;7:e32844. 10.1371/journal.pone.0032844 22393453 PMC3290602

[17] Henry J, Pylypchuk Y, Searcy T. Adoption of electronic health record systems among U.S. Non-Federal acute care hospitals, 2016: 2008–15. https://dashboard.healthit.gov/evaluations/data-briefs/non-federal-acute-care-hospital-ehr-adoption-2008-2015.php 40127322

[18] Kelly CJ, Karthikesalingam A, Suleyman M, et al. Key challenges for delivering clinical impact with artificial intelligence. BMC Med 2019;17:195. 10.1186/s12916-019-1426-2 31665002 PMC6821018

[19] Price WN, Nicholson Price IIW. Big data and black-box medical algorithms. Sci Transl Med 2018;10. 10.1126/scitranslmed.aao5333. [Epub ahead of print: 12 12 2018]. PMC6345162 30541791

[20] Gianfrancesco MA, Tamang S, Yazdany J, et al. Potential biases in machine learning algorithms using electronic health record data. JAMA Intern Med 2018;178:1544–7. 10.1001/jamainternmed.2018.3763 30128552 PMC6347576

[21] Hogan AH, Brimacombe M, Mosha M, et al. Comparing artificial intelligence and traditional methods to identify factors associated with pediatric asthma readmission. Acad Pediatr 2022;22:1–7. 10.1016/j.acap.2021.07.015 34329757

[22] Filipow N, Davies G, Main E, et al. Unsupervised phenotypic clustering for determining clinical status in children with cystic fibrosis. Eur Respir J 2021;58:2002881. 10.1183/13993003.02881-2020 33446607

[23] Howrylak JA, Fuhlbrigge AL, Strunk RC, et al. Classification of childhood asthma phenotypes and long-term clinical responses to inhaled anti-inflammatory medications. J Allergy Clin Immunol 2014;133:1289–300. 10.1016/j.jaci.2014.02.006 24892144 PMC4047642

[24] Farion KJ, Wilk S, Michalowski W, et al. Comparing predictions made by a prediction model, clinical score, and physicians: pediatric asthma exacerbations in the emergency department. Appl Clin Inform 2013;4:376–91. 10.4338/ACI-2013-04-RA-0029 24155790 PMC3799208

[25] Raita Y, Camargo CA, Bochkov YA, et al. Integrated-omics endotyping of infants with rhinovirus bronchiolitis and risk of childhood asthma. J Allergy Clin Immunol 2021;147:2108–17. 10.1016/j.jaci.2020.11.002 33197460 PMC8116357

[26] Sills MR, Ozkaynak M, Jang H. Predicting hospitalization of pediatric asthma patients in emergency departments using machine learning. Int J Med Inform 2021;151:104468. 10.1016/j.ijmedinf.2021.104468 33940479

[27] Huffaker MF, Carchia M, Harris BU, et al. Passive nocturnal physiologic monitoring enables early detection of exacerbations in children with asthma. A proof-of-concept study. Am J Respir Crit Care Med 2018;198:320–8. 10.1164/rccm.201712-2606OC 29688023 PMC6835062

[28] Shin EK, Mahajan R, Akbilgic O, et al. Sociomarkers and biomarkers: predictive modeling in identifying pediatric asthma patients at risk of hospital revisits. NPJ Digit Med 2018;1:50. 10.1038/s41746-018-0056-y 31304329 PMC6550159

[29] Seol HY, Shrestha P, Muth JF, et al. Artificial intelligence-assisted clinical decision support for childhood asthma management: a randomized clinical trial. PLoS One 2021;16:e0255261. 10.1371/journal.pone.0255261 34339438 PMC8328289

[30] Bose S, Kenyon CC, Masino AJ. Personalized prediction of early childhood asthma persistence: a machine learning approach. PLoS One 2021;16:e0247784. 10.1371/journal.pone.0247784 33647071 PMC7920380

[31] Wang AL, Datta S, Weiss ST, et al. Remission of persistent childhood asthma: early predictors of adult outcomes. J Allergy Clin Immunol 2019;143:1752–9. 10.1016/j.jaci.2018.09.038 30445065 PMC7061344

[32] Messinger AI, Bui N, Wagner BD, et al. Novel pediatric-automated respiratory score using physiologic data and machine learning in asthma. Pediatr Pulmonol 2019;54:1149–55. 10.1002/ppul.24342 31006993 PMC6641986

[33] Goto T, Camargo CA, Faridi MK, et al. Machine Learning-Based prediction of clinical outcomes for children during emergency department triage. JAMA Netw Open 2019;2:e186937. 10.1001/jamanetworkopen.2018.6937 30646206 PMC6484561

[34] Patel SJ, Chamberlain DB, Chamberlain JM. A machine learning approach to predicting need for hospitalization for pediatric asthma exacerbation at the time of emergency department triage. Acad Emerg Med 2018;25:1463–70. 10.1111/acem.13655 30382605

[35] Ross MK, Yoon J, van der Schaar A, et al. Discovering pediatric asthma phenotypes on the basis of response to controller medication using machine learning. Ann Am Thorac Soc 2018;15:49–58. 10.1513/AnnalsATS.201702-101OC 29048949 PMC5822415

[36] Das LT, Abramson EL, Stone AE, et al. Predicting frequent emergency department visits among children with asthma using EHR data. Pediatr Pulmonol 2017;52:880–90. 10.1002/ppul.23735 28557381

[37] Luo G, Stone BL, Fassl B, et al. Predicting asthma control deterioration in children. BMC Med Inform Decis Mak 2015;15:1–8. 10.1186/s12911-015-0208-9 26467091 PMC4607145

[38] van Horck M, Smolinska A, Wesseling G, et al. Exhaled volatile organic compounds detect pulmonary exacerbations early in children with cystic fibrosis: results of a 1 year observational pilot study. J Breath Res 2021;15:026012. 10.1088/1752-7163/abda55 33630756

[39] Pité H, Gaspar Ângela, Morais-Almeida M. Preschool-Age wheezing phenotypes and asthma persistence in adolescents. Allergy Asthma Proc 2016;37:231–41. 10.2500/aap.2016.37.3955 27074977

[40] Spyroglou II, Spöck G, Rigas AG, et al. Evaluation of Bayesian classifiers in asthma exacerbation prediction after medication discontinuation. BMC Res Notes 2018;11:522. 10.1186/s13104-018-3621-1 30064478 PMC6069881

[41] Robroeks CM, van Berkel JJ, Jöbsis Q, et al. Exhaled volatile organic compounds predict exacerbations of childhood asthma in a 1-year prospective study. Eur Respir J 2013;42:98–106. 10.1183/09031936.00010712 23645402

[42] Lovrić M, Banić I, Lacić E, et al. Predicting treatment outcomes using Explainable machine learning in children with asthma. Children 2021;8:376. 10.3390/children8050376 34068718 PMC8151683

[43] Van Vliet D, Smolinska A, Jöbsis Q, et al. Association between exhaled inflammatory markers and asthma control in children. J Breath Res 2016;10:016014. 10.1088/1752-7155/10/1/016014 26893372

[44] Caparrós-Martín JA, Flynn S, Reen FJ, et al. The detection of bile acids in the lungs of paediatric cystic fibrosis patients is associated with altered inflammatory patterns. Diagnostics 2020;10:282. 10.3390/diagnostics10050282 PMC7277992 32384684

[45] Khasha R, Sepehri MM, Mahdaviani SA. An ensemble learning method for asthma control level detection with Leveraging medical knowledge-based classifier and supervised learning. J Med Syst 2019;43:158. 10.1007/s10916-019-1259-8 31028489

[46] Moons KGM, Wolff RF, Riley RD, et al. PROBAST: a tool to assess risk of bias and applicability of prediction model studies: explanation and elaboration. Ann Intern Med 2019;170:W1–33. 10.7326/M18-1377 30596876

[47] Sounderajah V, Ashrafian H, Rose S, et al. A quality assessment tool for artificial intelligence-centered diagnostic test accuracy studies: QUADAS-AI. Nat Med 2021;27:1663–5. 10.1038/s41591-021-01517-0 34635854

[48] Sendak MP, Arcy JD, Kashyap S. A path for translation of machine learning products into healthcare delivery. EMJ Innov 2020.

[49] Seneviratne MG, Shah NH, Chu L. Bridging the implementation gap of machine learning in healthcare. BMJ Innov 2020;6:45–7. 10.1136/bmjinnov-2019-000359

[50] Char DS, Shah NH, Magnus D. Implementing machine learning in health care — addressing ethical challenges. N Engl J Med 2018;378:981–3. 10.1056/NEJMp1714229 29539284 PMC5962261

[51] Pinsky P. Electronic health records and machine learning for early detection of lung cancer and other conditions: thinking about the path ahead. Am J Respir Crit Care Med 2021;204:389–90. 10.1164/rccm.202104-1009ED 34097833 PMC8480236

[52] Benjamens S, Dhunnoo P, Meskó B. The state of artificial intelligence-based FDA-approved medical devices and algorithms: an online database. NPJ Digit Med 2020;3:118. 10.1038/s41746-020-00324-0 32984550 PMC7486909

[53] Muehlematter UJ, Daniore P, Vokinger KN. Approval of artificial intelligence and machine learning-based medical devices in the USA and Europe (2015–20): a comparative analysis. Lancet Digit Health 2021;3:e195–203. 10.1016/S2589-7500(20)30292-2 33478929

[54] Bica I, Alaa AM, Lambert C, et al. From real-world patient data to individualized treatment effects using machine learning: current and future methods to address underlying challenges. Clin Pharmacol Ther 2021;109:87–100. 10.1002/cpt.1907 32449163

[55] Adibi A, Sadatsafavi M, Ioannidis JPA. Validation and utility testing of clinical prediction models: time to change the approach. JAMA 2020;324:235–6. 10.1001/jama.2020.1230 32134437

[56] Albert RK. Informing healthcare decisions with observational research assessing causal effect: an American thoracic Society statement not ready for implementation. Am J Respir Crit Care Med 2021;204:374–6. 10.1164/rccm.202102-0492LE 34081880 PMC8513590

[57] Smith H. Clinical AI: opacity, accountability, responsibility and liability. AI Soc 2021;36:535–45. 10.1007/s00146-020-01019-6

[58] Wilkinson J, Arnold KF, Murray EJ, et al. Time to reality check the promises of machine learning-powered precision medicine. Lancet Digit Health 2020;2:e677–80. 10.1016/S2589-7500(20)30200-4 33328030 PMC9060421

[59] Chen IY, Pierson E, Rose S, et al. Ethical machine learning in healthcare. Annu Rev Biomed Data Sci 2021;4:123–44. 10.1146/annurev-biodatasci-092820-114757 34396058 PMC8362902

[60] Wiens J, Saria S, Sendak M, et al. Do no harm: a roadmap for responsible machine learning for health care. Nat Med 2019;25:1337–40. 10.1038/s41591-019-0548-6 31427808

[61] Goldstein BA, Navar AM, Pencina MJ, et al. Opportunities and challenges in developing risk prediction models with electronic health records data: a systematic review. J Am Med Inform Assoc 2017;24:198–208. 10.1093/jamia/ocw042 27189013 PMC5201180

